# How to Improve Pancreatic Cancer Network Care Using a Human-Centered Design Sprint

**DOI:** 10.2196/55598

**Published:** 2025-09-05

**Authors:** Jana S Hopstaken, Mats Koeneman, Robin Hooijer, Concha C van Rijssel, Theo van Voorthuizen, Frank A Oort, Charlotte F J M Blanken, Martijn de Groot, Cees J H M van Laarhoven, Martijn W J Stommel

**Affiliations:** 1 Department of Surgery, Radboudumc, Geert Grooteplein 10 (route 618), Nijmegen, 6525 GA, The Netherlands, 31 0243668086; 2 Health Innovation Labs, Radboudumc, Nijmegen, The Netherlands; 3 Department of Internal Medicine, Rijnstate, Arnhem, The Netherlands; 4 Department of Gastroenterology, Rijnstate, Arnhem, The Netherlands; 5 Department of Surgery, Rijnstate, Arnhem, The Netherlands

**Keywords:** pancreatic cancer, network care, continuity of care, human-centered design, patient care, continuous care, patient, expert, stakeholder, interview, experience, prototype, care, diagnosis, treatment, real-time, implementation, Netherlands, surgeon, oncologist, gastroenterologist, clinician, pancreatic, eHealth

## Abstract

Pancreatic cancer is considered a complex cancer requiring specific expertise in diagnostic workup and multimodality treatment. Often, multiple health care providers in different hospitals are involved during patient care. This fragmentation of care challenges health care providers in the network to deliver efficient, coherent, and continuous network care. We performed a human-centered design (HCD) sprint in order to find means to improve network care for patients diagnosed with pancreatic cancer. The sprint comprised 5 days with different goals: empathize, define, ideate, prototype, and test. Experts and stakeholders were approached from the pancreatic cancer network to contribute. By using HCD, a goal was defined, various prototypes were explored, and one prototype was tested. The HCD Sprint led to a shared goal, which was to deliver pancreatic cancer network care in a (virtual) hospital in which there is “one narrative.” This means that the patient’s context and preferences are always clear and taken into account, care is characterized by a short time to diagnosis and treatment, and patient data are easily available for patients and involved clinicians. The accompanying prototypes were (1) network agreements, (2) patient itinerary, (3) transmural trajectory guidance, and (4) data sharing. For the latter, we developed and pilot-tested a real-time data sharing dashboard called CONNECT. The first pilot-test was promising and provided feedback for further development. In this viewpoint paper, we show that a HCD sprint is able to find possible means to improve pancreatic network care in a short time span. A real-time data sharing dashboard (CONNECT) was developed and pilot tested. The next steps include further development of the dashboard, implementation in our network, and long-term evaluation studies.

## Introduction

Over the past decades, oncological health care services have changed considerably. For many types of cancer, the increasing complexity of care has led to the centralization of care. Centralization refers to the policy in which (parts of) oncological care, such as surgery, is concentrated to expert centers or high-volume hospitals. The main aims for centralization are improved quality of care and patient outcomes, better use of resources, and cost reduction [1]. In the Netherlands, centers that do not meet the volume standards for certain cancer operations, the non-expert centers, are required to refer patients to expert centers for treatment advice and surgery. However, these non-expert centers, or referring hospitals, play a large role in the diagnostic workup and provision of other parts of care, such as adjuvant chemotherapy or supportive care. The expert center together with the non-expert centers form a cancer network. In many European countries, these types of cancer networks can recognize tumors of the pancreas [2,3], liver [4], esophagus and stomach [5,6], ovaries [7,8], and head and neck [9]. Although the centralization of pancreatic surgery has provided better oncological care in terms of improved postoperative outcomes [10-13], it has resulted in fragmented cancer care delivery. Patients diagnosed with pancreatic cancer visit both non-expert and expert centers in their patient journey to consult with different medical specialists. This poses a challenge for patients but also for health care professionals. A recent, qualitative Dutch study investigating the experiences of patients diagnosed with pancreatic cancer, their relatives, and health care professionals showed that miscommunication between health care providers and the logistics between hospitals was the main cause of energy drain for patients in their patient journey [14]. The interviewed health care professionals reported challenges of logistics, inconsistencies in the information disclosed to patients, and the lack of overview of the patient. Regarding quality parameters of oncological care, multicenter care in Dutch pancreatic cancer patients is associated with repeated diagnostic investigations, delayed time-to-diagnosis, and delayed time-to-treatment [15]. These studies illustrate the complexity of pancreatic cancer network care and underline the necessity of close collaboration between the non-expert and expert centers.

Design thinking, or human-centered design (HCD), is increasingly recognized as an effective method for addressing complex problems, with recent research supporting its applicability in the field of health care in particular [16-19]. In HCD, the needs of persons are translated into a design that is technologically feasible and has value for its users. A systematic review indicated that HCD requires less time and may result in more effective, usable, and acceptable interventions compared to traditional methods of problem-solving [16]. The key strengths are its human-centered approach and its value in cross-disciplinary problem-solving, emphasizing diverse teams and iterative ideation. Moreover, the emphasis on prototyping and experimentation aligns with the principles of innovation and adaptability, essential qualities in the context of health-related challenges [20]. Examples of innovations or solutions of problems in health care that were approached with HCD are for instance improved nursing handoffs [21], clinical decision support for patients with hypertension and chronic kidney disease [22], new prenatal care models [23], and web-based platforms for patients with leukemia [24]. In this viewpoint paper, we demonstrate how we were able to find means to improve pancreatic cancer network care, by applying a HCD methodology.

## How We Performed the HCD Sprint

### Setting and Context

Between July 1, 2020 and July 10, 2020, we carried out a research project based on the principles of HCD. This project took place in the Radboudumc, Nijmegen, the Netherlands. The Radboudumc is a pancreatic expert center with six affiliated referring, non-expert centers. From this network, stakeholders were selected. The selection of the stakeholders was done by means of purposive sampling, taking into account representation of the key specialisms from the expert center and the largest non-expert center of the network, as well as variation in age and gender, and influential positions within the centers. Stakeholders that were involved were two surgeons (MS and CB), two medical oncologists (TvV), one gastroenterologist (FO), one medical case-manager, a professor of oncology networks, one oncology care manager, four members of the innovation team (MK, RH, CvR, and MdG), an IT-expert, a financial expert, and a family member of a patient with pancreatic cancer. Some clinicians were working in the expert center, and some in the non-expert center. During the design sprint, one of the members of the innovation team took charge of explaining the design sprint and tasks. This innovation expert (CvR) was also a moderator. Additionally, a Decider (MS) was appointed. During the design sprint, decisions had to be made by the involved stakeholders. In case of an undecided vote, the vote of the Decider (MS) was decisive.

### A HCD Sprint

HCD, interchangeable with terms such as design thinking or user-centered design, is a step-by-step, iterative process that aims to solve fundamental questions, a business problem, or issues in an organization [25]. One way to execute HCD in a short dedicated period of time is by performing a Design Sprint. The Design Sprint methodology is a framework, initially developed at Google, that has a 5-day approach aimed at tackling critical questions by rapidly designing, prototyping, and testing ideas with users [26]. The condensed time span is convenient for medical professionals that need to become involved, as it is challenging to combine clinical duties with a long-term project.

**Figure 1.**
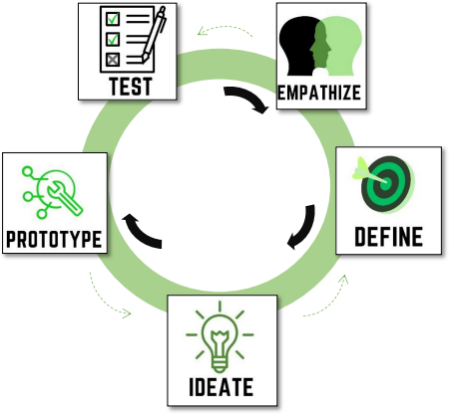
Steps of human-centered design (the design sprint for each phase is a day).

The design sprint is characterized by the phases: (1) Empathize, (2) Define, (3) Ideate, (4) Prototype, and (5) Test (Figure 1) [27]. These phases are non-linear and are carried out iteratively.

Prior to the sprint, we performed semi-structured interviews with 3 patients with pancreatic cancer, 1 family member, and 4 clinicians. The goal was to identify needs of patients and their relatives. Additionally, the goal was to gain a better understanding of the experiences of patients and the involved clinicians during network care. This served as input for the first phase of the design sprint. The yield of the semi-structured interviews with patients and their relatives prior to the design sprint was the starting point of phase 1. There was a clear need for improved continuity of care through improved communication between patients and medical specialists from different hospitals and improved communication between the medical specialists within the network.

## What We Learned from the HCD Sprint

### Day 1: Empathize

On day 1, all stakeholders visited the innovation laboratory at our center. After a short introduction, they exchanged views and needs on the current pancreatic cancer network care. The problem of care fragmentation within the pancreatic network and the need for a solution were introduced by the surgeon from the pancreatic expert center (MS). The stakeholders performed exercises that required them to clearly formulate what the ultimate goal would be. What needs to be there in order for network care to be a success? As soon as the goal and accompanying questions were clearly defined, a map was drawn. Every person in the team wrote sticky notes that indicated how this goal might be reached: the How Might We (HMW) notes. The HMW notes describe a mean or possible solution to reach a certain goal. HMW notes were stuck to a wall and organized into groups. The HMW notes were then prioritized by voting by the stakeholders. The voted HMW notes were placed within the map.

For the exercises, the stakeholders started off with a “Headline for the future” exercise. All stakeholders had to formulate a newspaper headline with accompanying text or illustration that would be published 4 years from now. Headlines that were drawn up were: “Costs of pancreatic cancer care diminished drastically by easy patient data exchange tool in hospitals,” “Regional pancreatic cancer network delivers continuity of care by joint effort with 7 hospitals,” or “Together stronger against pancreatic cancer by increasing collaboration between centers.” After discussion of these future headlines and voting, a goal of this sprint was formulated (Textbox 1).

**Textbox 1. Goal of this design sprint.**

To deliver pancreatic cancer network care in a (virtual) hospital in which:

- there is ‘one main entrance’ and ‘one narrative’
- the patient’s context and preferences are always clear and taken into account
- care is characterized by a short time to diagnosis and treatment, improved patient satisfaction, and survival
- patient data are easily available for patients and for involved medical professionals

After this formulation, the stakeholders had to formulate possible barriers in reaching this goal. They came up with different barriers. These were written down on sticky notes and placed on a board within the room. All barriers could be categorized under “Finance,” “Collaboration,” “Patient centeredness,” “IT,” “Priority and Focus,” or “Legal regulations.”

Of these themes, the team formulated the necessary conditions for the goal to be reached: (1) priority of this goal within health care organizations, (2) financial aids, (3) equality in expertise and knowledge from the involved clinicians towards the patient, and (4) arrangement to share patient data within contemporary national and European legal frameworks.

**Figure 2.**
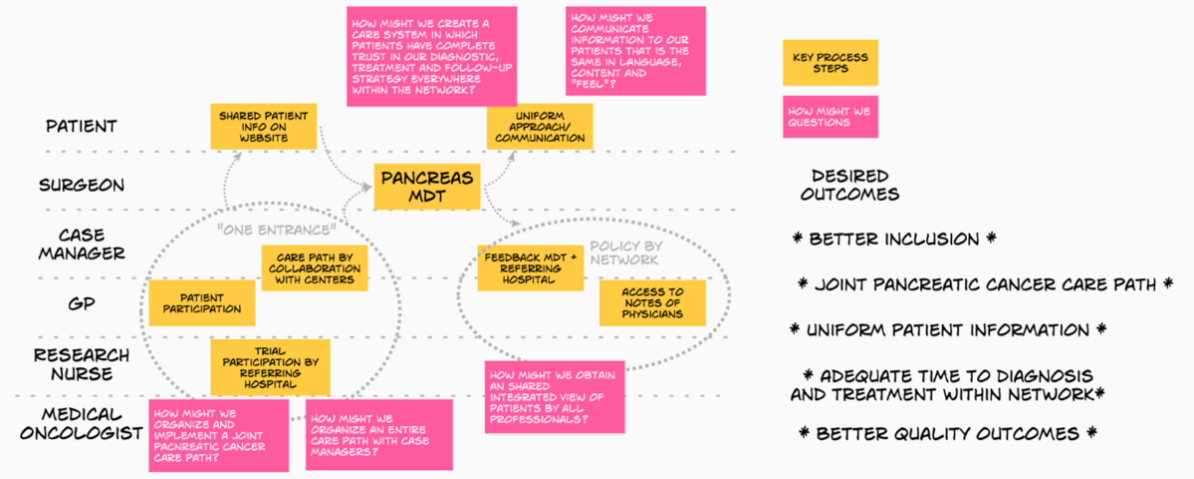
Map with patient care process and five “how might we” notes.

Then, the team further explored the problem. A map was drawn with the stakeholders of the pancreatic cancer network on the left side and the ultimate goals on the right side. This map was presented to two next of kin of patients. They were invited to add suggestions and questions. Subsequently, the stakeholders formulated the HMW notes. More than 80 HMW notes were formulated. Each team-member voted on the most promising, which eventually led to the election of 5 HMW notes (Figure 2).

### Day 2: Define

On day 2, all stakeholders regrouped and evaluated the results of day 1. These results were processed, reiterated, and eventually used to formulate a problem statement. In addition to this broad goal, desirable outcomes were formulated. These were (1) an equal pancreatic cancer care pathway, (2) uniform information towards patients, (3) short time to diagnosis and time to treatment within the pancreatic cancer network, (4) improved patient outcomes, and (5) better inclusion of patients in clinical trials.

### Day 3: Ideate

In the Ideate phase, the stakeholders concentrated on the generation of ideas that could potentially help to the goal that was formulated on day 2. To stimulate creativity and the thinking process, several exercises, such as the Lightning Demo and The Crazy 8s, were carried out [26]. The Lightning Demo is an exercise in which each stakeholder searches for existing ideas that could serve as an inspiration to solve the problem. Everyone creates a list of ideas, products or inventions. All stakeholders started with the Lightning Demo. The central question here was, “How do these examples provide ‘one entrance’ for their consumers to an organization or deliver a policy that is supported by different stakeholders or centers?” Twenty-one demos were formalized. Among these were Booking.com, Amazon, and the travel app of the Dutch Railway. After the Lightning Demo, the stakeholders started with the Crazy 8s exercises. The Crazy 8s is a fast-paced exercise in which each stakeholder takes their strongest ideas and sketches 8 variations of this idea in 8 minutes. Not all of these ideas are useful; some may even be impractical or impossible, but they give way for inspiring ideas and one or two may be the starting point of the great solution. The so-called solution sketches are displayed on board.

**Figure 3.**
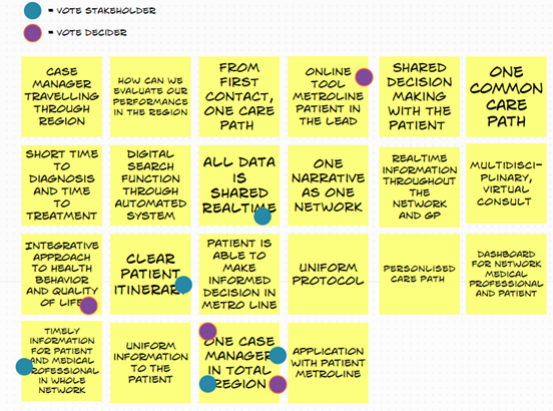
Most important elements that need to become incorporated in the prototype.

Subsequently, each stakeholder was able to vote on the solution sketch deemed most useful or on elements of the solution sketch. The team-members who made these solution sketches were asked to pitch these solutions (Figure 3). Afterwards, the most important elements of the sketches were drawn up and votes were provided. Elements with the most votes, together with the vote of the Decider (MS), would become incorporated in the prototype.

### Day 4: Prototype

**Figure 4.**
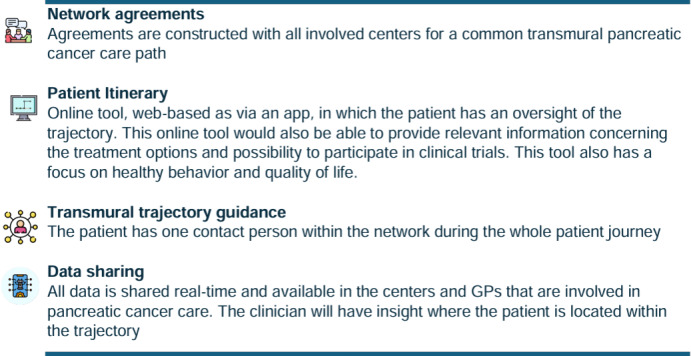
Four elements of the solution sketches

**Figure 5.**
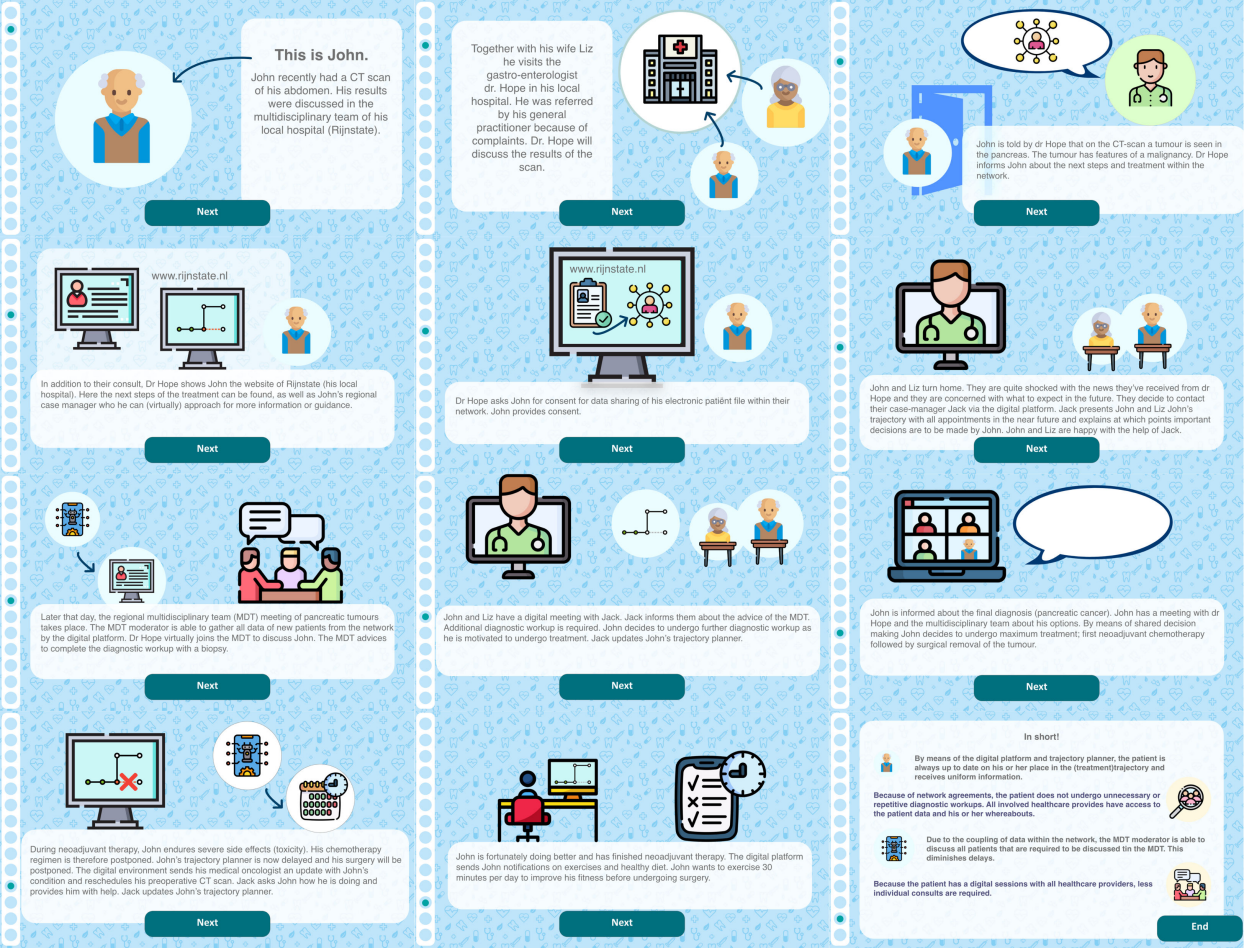
*Envisioned patient journey of a patient diagnosed and treated with pancreatic cancer in a pancreatic cancer network (From left to right)*.

**Figure 6.**
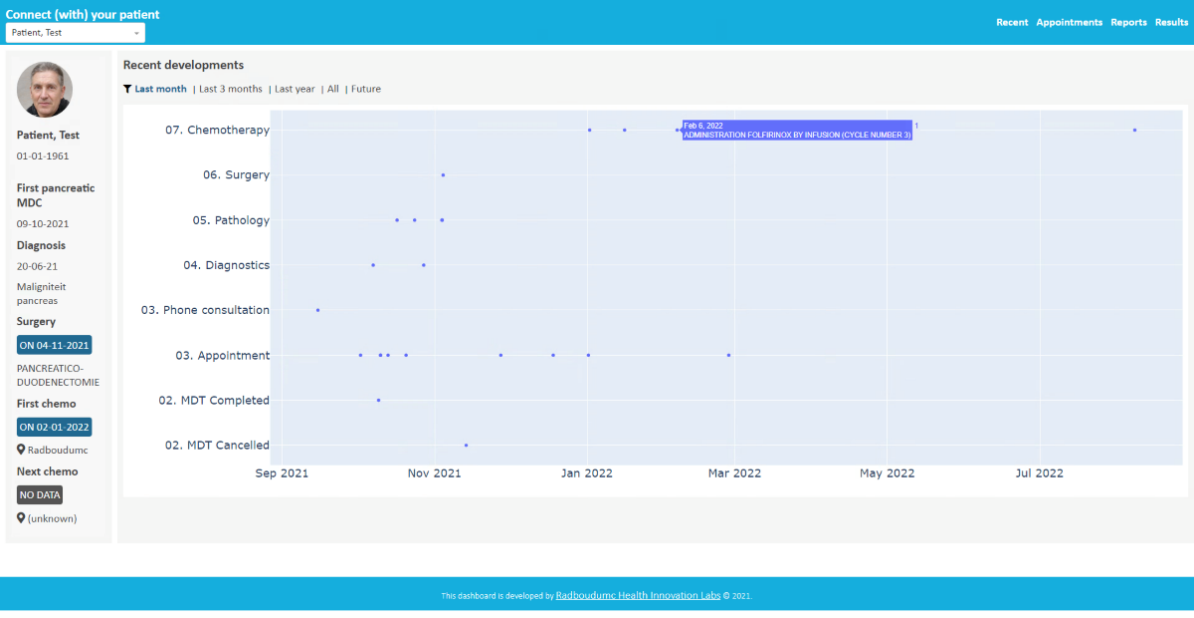
Prototype of “CONNECT”, an open-source community dashboard that enables real-time insight in patient information and the patient trajectory throughout the network.

In the prototype phase, the stakeholders constructed a prototype. This could be anything as long as it was interactive, for example, a storyboard, print screens of apps, or sketches of the solution. In the early phase of the prototype development, it is important to create prototypes that are quickly produced and affordable. However, they do need to be created as such that they can elicit feedback by the stakeholders or possible users. This allows for testing and feedback. The primary objective of prototyping is to gather feedback, learn from users, and refine the design based on their insights. Four elements of the solution sketches that were deemed most important were identified (Figure 4). We integrated these elements in a patient journey of a patient diagnosed with pancreatic cancer in the pancreatic cancer network (Figure 5). Each of these elements were further developed in a prototype. For the testing phase, we focused on one of these four elements; the real-time sharing of data through Dashboard “Connect,” an open-source community (Figure 6).

### Day 5: Test

The prototype was developed and was tested by the intended users. Testing allows to understand the end-users better and to further adjust the prototype to the users’ needs. Testing may lead to adjustments to the prototype but may also reveal that the problem was misframed. This would therefore lead to further iteration. Iteration is paramount for a good design [27]. In our HCD sprint, a prototype, a demo version of a real-time data sharing dashboard, was tested for a period of 8 weeks. The intended users were medical professionals outside our center who were involved in the pancreatic cancer care network. Three physicians were approached and were enabled to access the patient file of the expert center, allowing for a complete overview of patient information prior to the patient visiting their center. After a period of 8 weeks, the physicians were interviewed to evaluate their experiences. They were asked how much they used the dashboards to access the patient file and were asked questions concerning user-friendliness, accessibility, clarity, feasibility, and if they indeed had more insight in the patient trajectory throughout the network.

Some struggles using the dashboard were identified:

*Sometimes I forgot my password to log on to the system. But this happened only a few times.*

*I saw a patient at my outpatient clinic for whom I thought it would have been useful to use the dashboard. I knew the patient recently had an MRI at the expert center and wanted to know the results. However, I was not able to see whether the patient had provided consent for data exchange and I could use the dashboard for this patient. In the end, I called the case manager for an update.*

Other responses suggested that the dashboard had additional value and met the needs of its users:

*Prior to my consult with the patient, I was able to read the multidisciplinary team report and what the surgeon in the pancreatic expert center had discussed with the patient. I thought this was useful.*

*We receive information through letters, but sometimes the decision-making process or trade-offs are not described. By using the dashboard, I was able to see the notes made by the involved clinicians and was able to better understand the decision-making process.*

I think the possibility to have insight in patient information from the other center through the dashboard has additional value. (…) This way we can improve patient care.

For the next steps, we believe it is beneficial to perform multiple HCD sprints, involving a diverse mix of stakeholders, enhancing the proof-of-concept prototype’s rigor. Insights from multiple sprints could inadvertently enhance the development of the dashboard. We deemed one design sprint to be good enough to develop a first prototype and work with that as a learning tool rather than invest in more design sprints. If a repeated HCD sprint leads to a similar prototype, we aim to further explore this prototype and acquire funding for the development of the dashboard.

## Discussion and Future Directions

In this viewpoint article, we demonstrated that a HCD sprint may enable health care providers to find means to improve pancreatic cancer network care. The aim formulated by the involved stakeholders was to “deliver pancreatic cancer network care in a (virtual) hospital in which there is one main entrance and one narrative, the patient context and preferences are always clear and taken into account, care is characterized by a short time to diagnosis and treatment, patient satisfaction and survival benefit and where patient data are easily available for both patients and involved medical professionals.” The key elements to reach this goal were identified during our sprint and included network agreements, a clear patient itinerary, transmural trajectory guidance, and real-time sharing of data throughout the network. In our testing phase, we focused on real-time sharing of data throughout the network. The proof-of-concept prototype that was developed enabled safe and real-time insights in patient files between multiple hospitals and was evaluated as positive by the interviewed medical professionals. The other key elements to improve network care were addressed outside this sprint. Transmural trajectory guidance was provided through a case manager. A clear patient itinerary was developed by colleagues outside our project group [28]. Transmural agreements within our network concerning pancreatic cancer care were still under development.

To our knowledge, HCD sprints have not been used before in the improvement of pancreatic cancer network care. A different study on improving care coordination for patients with chronic diseases, in which a modified Delphi method was used to identify important points for improvement, indicated a few similar elements for improving care [29]. The authors recommended the development of measures of shared care between different professionals and health care systems. The development of data sharing dashboard, which was one of the outcomes that showed improvement in our project, would fit to this description. Additionally, this study recommended to make agreements concerning roles and responsibilities in the treatment process [29]. This is similar to one of the key elements delivered by our project: to construct transmural agreements within the network. Therefore, our results are to some extent in accordance with this study. Though this Delphi study indicated what stakeholders in such a network deem important, the Delphi study did not provide a clear solution or product to meet these needs and actually improve care coordination, as we were able to do by using a HCD. One of the pilots we developed was a dashboard that can share real-time data between institutions. Such a dashboard has been developed by the Belgian government, that is, the CoZo platform [30,31]. This platform was designed to facilitate communication and information exchange between health care providers, patients, and other stakeholders. It aims to improve coordination and continuity of care. While CoZo has been operational for several years, there are currently no studies that report on the long-term results. In literature, there are also a few other data-sharing dashboards but most of them are still in an early development phase, with observational studies being performed rather than studies concerning effectiveness [32,33].

Though the HCD method has positive aspects, it also carries some limitations. The HCD method has delivered valuable solutions and products for the tech industry but its robustness as a scientific method in health care is up for debate. HCD-based methods are unconventional in traditional biomedical research methods, as they are not a method to understand the nature of a phenomenon but more a strategy to find a solution to a problem, thus towards the invention of a new product, workflow, or protocol [19]. Traditional scientific methods should allow for reproducibility, falsifiability, and should be free from ideals. The HCD methodology is difficult to compare to the traditional scientific methodology [34]. Furthermore, the outcome of the HCD, that is the prototype, depends on the decisions made by the involved stakeholders. Group dynamics and type of stakeholders that were invited may shape the outcome of the HCD. Thus, a different group might have led to slightly different outcomes.

A strength of the HCD approach is that by inviting multidisciplinary stakeholders we were able to grasp different perspectives of current pancreatic network care, leading to a better understanding. This allowed us to target points for improvement and develop a solution that fits the needs of patients and involved clinicians. A second strength is that, in contrast to other studies [24,35-37], we chose to focus on improving the care process in the network by focusing more on input from clinicians. We underline the importance of patient-centered care and the patient’s ability to self-manage through platforms. However, to truly improve network care, it is equally important to study the experiences of the involved clinicians and to assess what they think is important to improve network care. With this study, we contribute to the knowledge of improving network care, from the perspective of the clinician. Third, we have shown, by using a HCD Sprint, that the first steps to changing and improving a current workflow does not require commitment of months or years, but may take only a week. Clinicians play a crucial role in improving health care, and we have shown with this HCD sprint that they can make a valuable contribution without the requirement to commit to a lengthy, time-consuming project. This approach, however, does require extensive preparation, guidance, and executional workforce from innovation facilitators.

The HCD sprint enabled us to formulate which elements of pancreatic network care are deemed as important points for improvement and has led to a prototype that may improve network care. Our findings may also be of value for other types of oncological network care for which there is centralization of surgery in place. Moreover, it is most likely that networks will increasingly become more widespread and implemented in health care systems that aim to efficiently use their resources. In the Netherlands, over a period of 4 years, the number of hospital alliances has doubled [38,39]. Second, the integrated health care agreement (IZA) published by the Dutch Ministry of Health also stated that in order to keep health care services affordable and of high-quality, collaboration between different clinicians and hospitals within networks is required [40]. A prerequisite for adequate network care is the easy and fast exchange of patient information, as was also formulated as a key element in our sprint. To facilitate this, the Dutch Government is currently constructing a Law on Electronic Data Exchange in Health Care, which enforces the exchange of electronic patient files under conditions that meet certain quality standards. Furthermore, the European Commission has made it a priority to facilitate easy and fast exchange of medical information (European Health Data Space). Achieving this goal will probably be challenged by privacy and safeguarding of patient data. Additionally, it is up for debate which party would be best appointed to develop such a platform. IT businesses may be able to successfully develop a platform that meets the requirements identified by its stakeholders but would require commitment from a significant number of hospitals as clients in order for the business case to be viable. Second, it would render hospitals dependent on the successes and whims of IT companies. Alternatively, an open-source approach, such as open electronic health records [41], may be able to develop a patient information exchange platform that is accessible for all hospitals to allow seamless network care.

## Conclusions

Pancreatic cancer network care is challenged by the fragmentation of care. By means of a HCD sprint, we found that possible means to improve pancreatic network care are network agreements, a clarified patient itinerary, transmural trajectory guidance, and improved patient information exchange and data sharing. Improved patient information exchange was prototyped and pilot-tested. The next step is to further develop our dashboard in order to improve pancreatic cancer network care.

## Abbreviations

HCD: human-centered design
HMW: How Might We
